# Counseling and Knowledge of Danger Signs of Pregnancy Complications in Haiti, Malawi, and Senegal

**DOI:** 10.1007/s10995-018-2563-5

**Published:** 2018-06-23

**Authors:** Shireen Assaf

**Affiliations:** ICF, The Demographic and Health Surveys (DHS) Program, 530 Gaither Road, Suite 500, Rockville, MD 20850 USA

**Keywords:** Antenatal care, Counseling, Provider observation, Client exit interview, Knowledge of danger signs, Pregnancy complications

## Abstract

*Objectives* Providing counseling on danger signs of pregnancy complications as part of visits for antenatal care (ANC) can raise expecting women’s awareness so that if danger signs occur they can seek assistance in time. The study examines the level of agreement in counseling on danger signs between observation of the provider during the ANC visit and the client’s report in the exit interview, and the association of this agreement with the client’s level of knowledge on danger signs. *Methods* The analysis used data from service provision and assessment (SPA) surveys in Haiti, Malawi, and Senegal. Agreement between the observation and client’s report was measured by Cohen’s kappa and percent agreement. Regressions were performed on the number of danger signs the client knew, with the level of agreement on the counseling on danger signs as the main independent variable. *Results* The study found little agreement between the observation of counseling and the client’s report that the counseling occurred, despite the fact that the exit interview with the client was performed immediately following the ANC visit with the provider. The level of positive agreement between observation and client’s report was 17% in Haiti, 33% in Malawi, and 23% in Senegal. Clients’ overall knowledge of danger signs was low; in all three countries the mean number of danger signs known was 1.5 or less. The regression analysis found that, in order to show a significant increase in knowledge of danger signs, it was important for the client to report that it took place. *Conclusions* Ideally, there should be 100% positive agreement that counseling occurred. To achieve this level requires raising both the level of counseling on danger signs of pregnancy complications and its quality. While challenges exist, providing counseling that is more client-centered and focuses on the client’s needs could improve quality and thus could increase the client’s knowledge of danger signs.

## Significance

Recognizing danger signs of pregnancy complications is essential for expecting mothers to identify early in order to seek assistance in time. Delays in seeking assistance may lead to adverse outcomes for the mother and child. This study examines the level of knowledge of expecting mothers in recognizing danger signs of pregnancy complications and the level and quality of counseling provided in health facilities of three countries. It uses observation of visits as well as the exit interviews for the analysis, and illustrates the need for better quality of counseling for increasing the already low levels of knowledge on danger signs of pregnancy complications in these countries.

## Introduction

Counseling during antenatal care (ANC) visits on danger signs of pregnancy complications is important because it can raise the awareness of expecting mothers in recognizing danger signs. However, improving ANC clients’ knowledge of danger signs of pregnancy complications depends not only on the amount of counseling provided but also on its effectiveness and on whether the information communicated by the provider can be easily understood and retained by the expecting mother. This study explores the level of knowledge and counseling on danger signs of pregnancy complications during ANC visits at health facilities in Haiti, Malawi, and Senegal. The outcome of interest is the number of danger signs women knew after their ANC visit. The main variable of interest is counseling on danger signs of pregnancy complications. We expect that receiving counseling would increase the women’s knowledge of danger signs.

Counseling provided during ANC visits has been shown to have an effect on improving several maternal and child health indicators, including delivery by a skilled birth attendant, birth preparedness, newborn care, and breastfeeding (Ahmad et al. 2012; Baqui et al. 2007; Dunlop et al. 2013; Mpembeni et al. 2007; Nikiéma et al. 2009). Counseling on danger signs that could lead to pregnancy complications can help pregnant women recognize these danger signs and seek assistance in a timely manner. Delays in seeking health care for pregnancy complications can increase the risk of maternal mortality and morbidity; recognizing and acting on danger signs of pregnancy complications can help reduce these risks (Duysburgh et al. 2013; Mbalinda et al. 2014).

For counseling to be effective in transferring knowledge to women during their ANC visits, it must be of good quality, and information must be successfully communicated by the provider and retained by the women. Having an effective counseling session is related to the provider’s technical competency, training, attitude toward counseling, and interpersonal relationship with the client during her visit, all of which can be linked to the quality of care provided (Bruce 1990; Donabedian 1988; Hutchinson et al. 2011; Vickers et al. 2007).

Measuring quality of care at health facilities often relies on either observations of provider-client interaction during consultations or client reports from exit interviews. Both types of information can be found in the service provision and assessment (SPA) surveys. The observations are performed during the client’s consultation visit with the health care provider at the facility. An exit interview is also performed with the client after the visit.

Examining the level of agreement between the observation of the consultation and the client’s report of the consultation in the exit interview can be valuable in understanding the quality of the care provided to the clients during any type of visit, including ANC. However, only a limited amount of research has assessed the consistency or agreement between observations and client reports, and especially for counseling provided during ANC visits. Two studies were found that compared observation and exit interviews (Bessinger and Bertrand 2001; Tumlinson et al. 2014), but these covered family planning visits rather than ANC visits. A broader study, from which this analysis originated, looked at the level of agreement between observation of providers and exit interviews with clients for several counseling items provided during ANC, family planning, and sick child care visits (Assaf et al. 2016). The study found low levels of agreement in most counseling topics.

The present analysis will examine the level of agreement between observation of providers and exit interviews with clients in counseling on danger signs of pregnancy complications during ANC visits and will assess the effect of this agreement on the knowledge of danger signs gained by the client.

## Methods

### SPA Data

The analysis used data from the SPA surveys conducted in Haiti in 2013 (Institut Haïtien de l’Enfance—IHE & ICF International 2014), Malawi in 2013–2014 (Ministry of Health-MoH/Malawi & ICF International 2014), and Senegal in 2014 (Agence Nationale de la Statistique et de la Démographie-ANSD/Sénégal & ICF International 2015). For Haiti and Malawi, the SPA surveys were a census, including all formal-sector health facilities in the country. For Senegal, the SPA survey was a sample of the country’s health facilities.

Data collected in the SPA surveys provide information on the health facility, including the infrastructure, equipment, and availability of medicines. A questionnaire is also administered to health providers to collect information on their characteristics, qualifications, and training. In addition, for a sample of providers the client’s visit is observed using an observation checklist—that is, a checklist of specific items the interviewer should observe during the health care visit. These provider observations and the client exit interviews are performed by trained interviewers, who are mostly health workers.

Clients visit a health facility to receive care for a number of reasons; however, in SPA surveys it is usually a visit for ANC, family planning, or child health care that is observed. Clients whose consultations are observed are automatically eligible for a client exit interview. The exit interview collects basic information about the client and on the visit with the provider. All surveys administered by the DHS Program (including SPA surveys) are reviewed and approved by the Institutional Review Board (IRB) and consent obtained from any interviewee before performing the interview or observation. More detailed information on SPA surveys and methodology can be found on the DHS Program website^1^. Table 1 shows the number of health facilities included in the three surveys covered by this study and the number of ANC consultations observed.

**Table 1:** Number of facilities and ANC observations included in the SPA surveys analyzed in this study

	Haiti 2013	Malawi 2013–2014	Senegal 2014
Number of facilities included in the SPA	905	977	363
Number of ANC consultations observed	1,620	2,068	1,211

### Measures

#### Counseling Variables

The ANC observation checklist contained several observations of counseling during the client’s visit. One of the counseling topics observed was on danger signs of pregnancy complications. The observation noted whether the provider mentioned any of seven main danger signs—vaginal bleeding; fever; swollen face or hands; tiredness or breathlessness; headache or blurred vision; cough or difficulty breathing; and reduced or no fetal movement.

During the counseling, the interviewer uses an observation checklist to note whether the provider asked about, advised, or discussed a specific topic with the client. The exit interview administered immediately following the ANC visit asks expecting mothers about their visit, including the counseling they received—specifically if the counseling was performed for this current visit, this and a previous visit, previous visit only, or not at all. In the analysis, the client’s response that counseling was performed for the current visit was used for a variable indicating that counseling on any of the danger signs was performed during the ANC consultation. The observation of the counseling and the client’s report were then combined for a variable with four categories: (1) both the observation and the client’s report agree that counseling was not provided (both no); (2) the provider was not observed to give the counseling but the client reported receiving it (prv no, cl yes); (3) the provider was observed to give the counseling but the client did not report receiving it (prv no, cl yes); and (4) both the observation and client’s report agree that the counseling was given (both yes). The first category of this variable can be considered a negative agreement and the fourth category as a positive agreement.

#### Knowledge of Danger Signs

During the exit interview clients were asked to state the danger signs of pregnancy complications that they know. The seven possible signs that the client could report are the same as in the observation checklist, except that the exit interview asked about “seizure or convulsions” instead of “cough or difficulty breathing.”

#### Independent Variables

The independent variables used in the analysis included characteristics related to the client, provider, and facility. The client’s variables included the client’s age, education (none, primary/post primary, or secondary or more), number of ANC visits (first visit, 2, 3, or 4 or more), and whether the pregnancy is their first. The provider variables included the occupational category (doctor/specialist/technician or nurse/midwife/other), years of education (< 16, 16–18, 19 or more), training in counseling within 24 months (yes or no), and the number of items on which they were supervised (none, 1–5, or 6). The facility-level independent variables included the managing authority (private/faith/NGO/other or government), facility type (hospital, health center, other, which includes health posts, health huts, etc.), urban or rural location, and region. For Haiti, regional departments were grouped into North (North, Northeast, and Northwest regions), Center (Artibonite and Center regions), South (South, Southeast, Grand-Anse, and Nippes regions), and West (West region). Malawi contained three regions (North, Center, and South). For Senegal, provinces were grouped as North (Louga, Matam, and Saint Louis regions), Dakar, Thiès, Center (Diourbel, Fatick, Kaffrine, and Kaokack regions), East (Kédougou and Tambacounda regions) and South (Kolda, Sédhiou, and Ziguinchor regions). In addition to these independent variables, the analysis also included the duration of the consultation in minutes.

### Analysis

Both Cohen’s kappa statistic and percent agreement were used to measure the level of agreement between observation of the counseling and the client’s report in the exit interview. A kappa of zero or less signifies no agreement, 0.01–0.20 is slight agreement, 0.21–0.40 is fair agreement, 0.41–0.60 is moderate agreement, 0.61–0.80 is substantial agreement, and 0.81–1.0 is perfect agreement (Viera and Garrett 2005).

The percent agreement is computed by dividing the number of clients who agreed with the observation that the counseling did or not occur by the total number of clients. A minimum agreement of 80% is considered acceptable (McHugh 2012). However, unlike the kappa statistic, we cannot know if this agreement is due to chance.

In the regression analysis the main outcome of interest was the number of danger signs the client reported from the possible seven signs they could report. Poisson or negative binomial regressions were used to fit the models depending on the result of a goodness of fit test for the Poisson model and a likelihood ratio test of the overdispersion parameter. Separate models were fit for all clients and for a subset of clients in their first pregnancy. Separate models were also fit for the variable that includes whether the client received counseling on danger signs of pregnancy complication and the variable that combines the observation of the counseling and the client’s report in the exit interview of whether they received counseling. Therefore, for each country in the analysis there are four models.

The analyses were performed at a 95% confidence level and accounted for the sample design and weights for each survey. For Haiti and Malawi, because the SPA was a census including all formal-sector health facilities in the country, no stratification was needed. For Senegal, because the SPA was a sample survey of the country’s health facilities, stratification was performed by facility type and region.

## Results

### Levels and Agreement on Counseling

As Table 2 shows, more than half of the ANC consultations in Haiti and Malawi were observed to provide counseling on danger signs, and just over a third in Senegal. In Haiti a significantly smaller proportion of women (30%) reported receiving the counseling, while in Senegal a significantly higher proportion (51%) reported receiving it. In Malawi there appears to be no significant difference between the observation of counseling and women’s report in the exit interview. However, in all three countries the percent agreement was less than the acceptable threshold of 80%, and the kappa statistic indicates only a slight agreement. The level of agreement was lowest in Haiti, higher in Senegal, and highest in Malawi. All the kappa estimates were significant, indicating that the slight agreement found is not due to chance.

**Table 2:** Percentage of observed counseling on danger signs of pregnancy complications and the percentage of clients that reporting receiving the counseling with reported percent agreement and kappa statistics

	Provider observed		Client reported		Percent agreement	Kappa
	%	CI	%	CI	%	
Haiti	51.0	(47.3, 54.6)	30.3	(26.8, 33.8)	52.3	0.053
Malawi	54.0	(49.3, 58.7)	51.3	(47.5, 55.1)	60.1	0.200
Senegal	38.0	(33.6, 42.3)	51.3	[(46.8, 55.9)	56.4	0.134

**Fig. 1:**
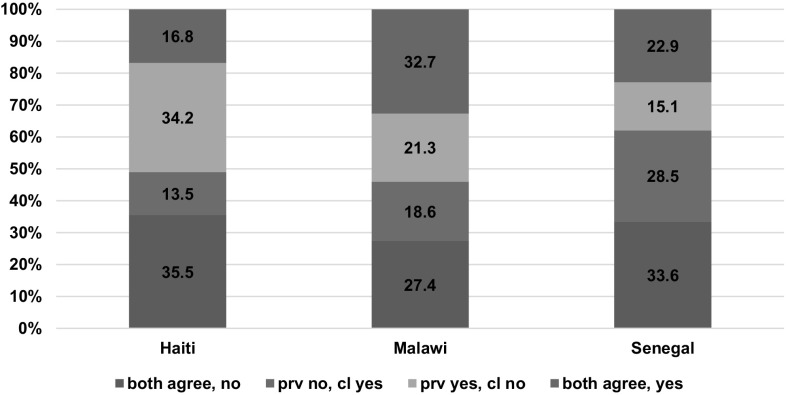
Agreement between the observation of counseling on danger signs and the client’s report in the exit interview. *Note* both agree, no: both the observation and client’s report agree; prov no, cl yes: provider was not observed to give the counseling but the client reports receiving the counseling; prov yes, cl no: the provider was observed to give the counseling but the client did not report receiving it; both agree, yes: positive agreement that the counseling did occur

Figure 1 shows the distribution of the variable on counseling for danger signs, which combines the observation and client’s report, in Haiti, Malawi, and Senegal. In accord with the kappa estimates in Table 2, the highest level of disagreement that the counseling occurred was in Haiti (48%), followed by Senegal (44%), and lowest Malawi (40%). The highest positive agreement that the counseling occurred was in Malawi (33%), followed by Senegal (23%), and lowest in Haiti (17%).

**Fig. 2:**
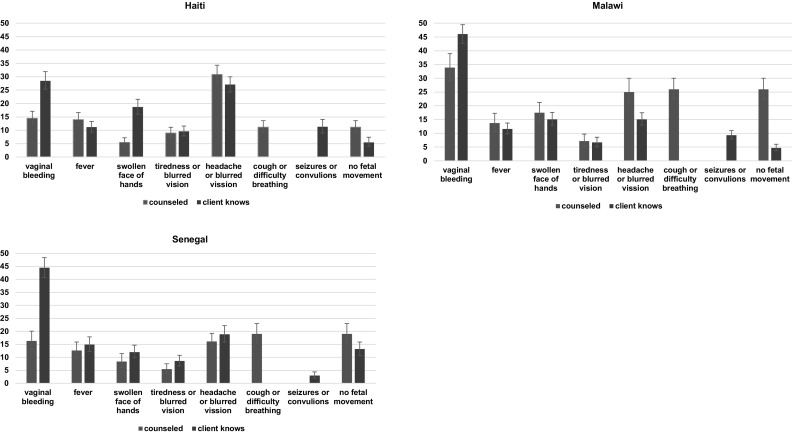
Counseling from provider and client knowledge of specific danger signs in Haiti, Malawi, and Senegal

In Fig. 2 we see the specific danger signs that the clients were counseled on and the specific danger signs that they reported knowing. Across all three countries, vaginal bleeding was the danger sign most known by clients, at almost 30% of clients in Haiti and 45% of clients in Malawi and Senegal. The item that was most counseled differed by country—in Haiti, headache or blurred vision (31%); in Malawi, vaginal bleeding (34%); and in Senegal, vaginal bleeding and headache or blurred vision (both 16%). Note that the danger sign of cough or difficulty breathing was one of the counseling items but was not one of the items that clients reported knowing. In addition, the seizures or convulsions danger sign was one of the signs clients reported knowing but was not one of the counseling items.

### Knowledge of Danger Signs

**Fig. 3:**
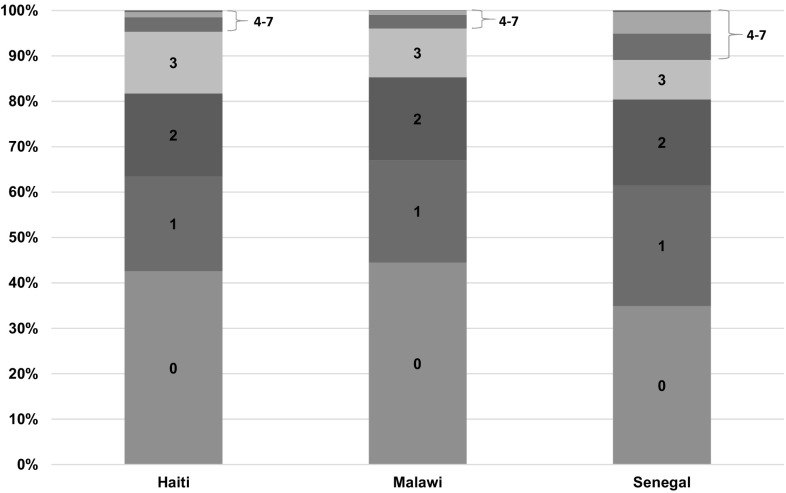
The number of danger signs reported by the client in the exit interview after the ANC visit

Figure 3 shows the distribution of the number of danger signs that clients reported from the total of seven possible signs. In all three countries, more than half of the clients knew at least one danger sign, but most clients could report only one, while almost no clients knew all seven.

**Fig. 4:**
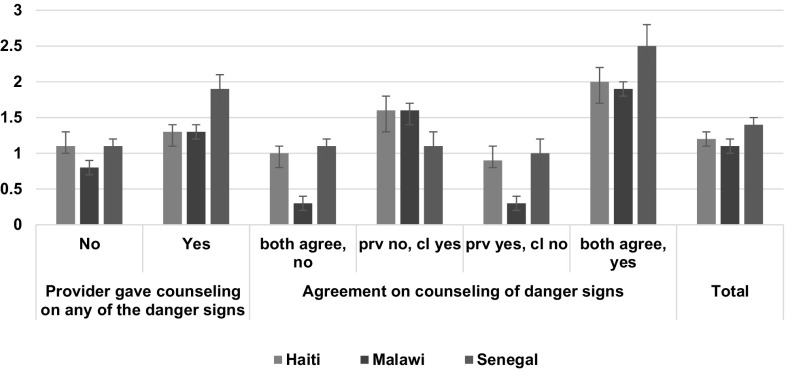
Mean number of danger signs reported by the client by the counseling variables. *Note* both agree, no: both the observation and client’s report agree; prov no, cl yes: provider was not observed to give the counseling but the client reports receiving the counseling; prov yes, cl no: the provider was observed to give the counseling but the client did not report receiving it; both agree, yes: positive agreement that the counseling did occur

Figure 4 shows the mean number of danger signs clients reported knowing, by the counseling variables. In all three countries, the mean number of danger signs known did not exceed 1.5. Clients who had positive agreement that counseling occurred reported a significantly higher number of danger signs compared with clients who agreed that the counseling did not occur. All three countries also showed no significant difference in the mean number of danger signs known between clients who reported that no counseling was provided but observation showed that counseling was given and clients who agreed with the observation that no counseling was given.

Table 3 summarizes the regression results of the number of danger signs that clients know, for Haiti, Malawi, and Senegal. Each country has four models: (1) a model for clients in their first pregnancy and with the binary variable of whether or not the provider was observed to give counseling; (2) the same as the first model but for all clients; (3) a model for clients in their first pregnancy and the counseling variable that combines the client’s report; and (4) the same as the third model but for all clients. All four models had a negative binomial distribution except for two models in Malawi that used the combined counseling variable. These two models followed a Poisson distribution.

**Table 3:** Regression of the number of danger signs that could occur during pregnancy that the client knows with reported incidence risk ratios

	Haiti				Malawi				Senegal			
Client type	1st Pregnancy		All clients		1st Pregnancy		All clients		1st Pregnancy		All clients	
Counseled on any of the danger signs (ref.=no)												
Yes	1.1		1.1		2.9***		1.6***		2.8***		1.7***	
Agreement on counseling on danger signs (ref.= bothno^a^)												
Prov no, cl yes^b^		1.8***		1.6***		7.4***		4.5***		1.1		1.0
Prov yes, cl no^c^		0.9		1.0		1.0		0.9		0.9		0.9
Bothyes^d^		2.4***		2.0***		11.9***		5.7***		3.8***		2.3***
Duration of consultation												
Average in mins	1.0	1.0	1.0	1.0	1.0	1.0	1.0	1.0	1.0	1.0	1.0**	1.0**
Client’s age (ref.: < 20)												
20–29	1.4*	1.4*	1.4**	1.3*	0.9	0.9	0.9	1.0	0.8	0.8	1.0	1.0
30–35	1.6**	1.5*	1.6***	1.5***	1.5	1.1	1.1	1.1	1.1	1.0	1.0	1.0
36+ and don’t know	1.2	1.3	1.6**	1.5**	0.8	0.7	1.0	1.1	1.3	1.1	1.1	1.1
Client’s education (ref.: none)												
Primary	1.0	1.1	1.3*	1.2	1.8	1.7	1.2*	1.2	1.4	1.4	1.1*	1.1*
Secondary +	1.2	1.2	1.4**	1.3**	3.2**	2.0*	1.6*	1.4*	1.9**	1.7**	1.3**	1.3*
Number of previous ANC visits (ref.: first visit)												
2	1.1	1.3	1.2**	1.3**	1.1	1.1	1.2	1.3***	1.2	1.2	1.2	1.2
3	1.1	1.2	1.3*	1.2*	1.1	1.2	1.2*	1.4***	1.7**	1.5*	1.2*	1.1
4 or more	1.2	1.1	1.3***	1.3**	1.4	1.3	1.3*	1.5***	2.6***	2.5***	1.5***	1.5***
Client’s first pregnancy (ref.: no)												
Yes			1.0	1.0			0.7***	0.9*			0.8**	0.7**
Provider years of education (ref.: < 16)												
16–18	0.9	1.0	0.9	0.9	0.7*	0.8*	1.0	0.9	0.8	0.7*	0.9	1.0
19+	0.8	0.9	0.8	0.9	0.5*	0.6**	0.8	0.8*	0.7	0.6*	1.0	1.0
Provider training in ANC counseling (ref.: no)												
Yes	0.9	0.8	0.9	0.9	0.9	1.2	1.0	1.1	1.4*	1.4	1.3***	1.2**
Provider number of items supervised (ref.: none)												
1–5	1.3	1.3	1.3*	1.3*	1.0	1.1	0.9	1.0	0.8	0.9	1.0	1.0
6	0.9	0.9	1.1	1.1	1.3	1.2	1.1	1.1	1.3	1.1	1.1	1.1
Region (ref.: North)												
Center	0.8	0.9	1.0	1.1	0.7	0.8	0.8	0.9				
South	1.1	1.2	1.1	1.1	0.6**	0.7*	0.7**	0.8*				
West	0.9	0.9	0.9	0.9								
Dakar									1.8	1.7	1.7**	1.8***
Thiès									1.4	1.1	1.8***	1.7***
Central									1.1	1.1	1.6***	1.6***
East									1.6	1.5	2.4***	2.5***
South									1.2	1.1	1.9***	1.8***
Observations	531	531	1,603	1,603	489	489	2,025	2,025	256	256	1,206	1,206

^a^Both no: both the observation and client’s report agree

^b^Prov no, cl yes: provider was not observed to give the counseling but the client reports receiving the counseling

^c^Prov yes, cl no: the provider was observed to give the counseling but the client did not report receiving it

^d^Both yes: positive agreement that the counseling did occur

All models control for provider category, facility managing authority, facility type and location, which were all not significant in the models. Significance: *p < 0.05, ** p < 0.001, *** p < 0.001

Table 3 shows that in Malawi and Senegal receiving counseling on danger signs of pregnancy complications significantly increased the number of danger signs the client knows. The incident risk ratios were higher for clients in their first pregnancy compared with all clients. In Haiti the binary counseling variable was not significant. When the combined counseling variable was used in the model, clients in Haiti showed a significant increase in the number of danger signs known only when they reported receiving the counseling. Malawi and Senegal showed the same findings, but in Senegal significance was only found when the client agreed with the observation that the counseling was given. For all three countries, there was no significant difference in the number of danger signs known between clients who agreed with the observation that no counseling was given and clients who were observed to be counseled but did not report being counseled. The findings were more or less the same for all clients as well as for clients in their first pregnancy. The significant incidence risk ratios were higher in the models that select for clients in their first pregnancy compared to models that include all clients. This was especially the case for the incidence risk ratios for the counseling variables.

## Discussion and Conclusions

This study examined the level and quality of counseling specifically on danger signs of pregnancy complications during client ANC visits to health facilities. The results show that approximately half of the clients in Haiti and Malawi received counseling on any of the danger signs of pregnancy complications, and in Senegal slightly more than a third of the clients. In addition, further analysis (not shown in the results) found that providers in Haiti and Senegal only counseled on an average of one danger sign, and in Malawi an average of 1.5 danger signs. These levels of counseling are insufficient, considering the importance of expecting mothers needing to recognize danger signs of pregnancy complications in order to seek assistance in time.

Hemorrhage and hypertensive disorders are considered to be the main causes of maternal deaths (Khan et al. 2006; Ronsmans et al. 2006). However, only approximately 15% of clients in Haiti, 16% of clients in Senegal, and 34% of clients in Malawi received counseling on vaginal bleeding as a danger sign (see Fig. 2). While vaginal bleeding was one of the danger signs most known by clients, less than half of clients in these countries reported that they knew this danger sign. Clients’ overall knowledge of danger signs was also low. In all three countries the mean number of danger signs known was less than 1.5. Low levels of counseling on danger signs of pregnancy complications and on clients’ knowledge of these danger signs have also been found in several other settings (Ali et al. 2010; Anya et al. 2008; Duysburgh et al. 2013; Jennings et al. 2010; Kabakyenga et al. 2011; Magoma et al. 2011; Mutiso et al. 2008; Nikiéma et al. 2009; Pembe et al. 2009).

The results show little agreement between the observation of counseling and the client’s report that the counseling occurred, despite the fact that the exit interview with the client was performed immediately following the ANC visit with the provider. Many factors could explain this low level of agreement. In Haiti significantly fewer clients reported receiving counseling than the observation of counseling showed was provided. This might be because clients did not pay attention to the counseling given. The counseling may also have been done quickly or not in a way that the client was able to understand. On the other hand, Senegal had significantly more clients reporting receiving counseling than observation showed was actually provided. In this case, clients might have reported receiving counseling even if it did not occur to avoid expressing dissatisfaction with services. Another explanation could be response bias, in which clients responded in a way they believed the interviewer expected—that they were counseled even when they knew they had not been. In cases such as Senegal, providing more counseling during ANC visits probably would increase agreement between observation and the exit interview. However, as in Haiti, when counseling took place but the client did not report receiving it, an improvement in the quality of the counseling would be needed to increase the level of agreement.

Providing a high quality of counseling can be a challenge. For ANC providers, the challenge is to improve counseling skills and receive training in effective, client-centered counseling. In Benin a randomized control study found that using job aids to deliver messages during ANC visits significantly increased the proportion of women with correct knowledge compared with a control group (Jennings et al. 2010). From the client’s perspective, receiving too much information can be overwhelming and there may be too many messages for the client to retain in the short time with the provider. The average duration of the ANC consultations in the three countries studied was less than 20 min, which may not be sufficient time to perform the ANC checkup and to offer effective ANC counseling. Providing a higher quality of counseling can take more time. In Tanzania a study found that implementing a new model for ANC visits that included a higher quality of counseling doubled the average duration of the visit (von Both et al. 2006). This would be a challenge to many providers who may only have a limited amount of time to spend with their clients, especially if several clients are waiting to be seen. Having few staff available to see clients would compound the problem and as a consequence decrease the amount of time available for the ANC visit, which could decrease the quality of the visit (Adam et al. 2005; Duffield et al. 2011; Igumbor et al. 2016). In fact, there has been a reported global crisis in the shortage of health care personnel, including in Haiti, Senegal, and Malawi (World Health Organization 2006).

A low level of knowledge in danger signs of pregnancy complications was also found to be associated with specific client, provider, and facility characteristics. For all three countries the number of danger signs known was significantly higher for clients with secondary or higher level of education versus no education. The exception was in Haiti, for the models that selected for clients in their first pregnancy. Having more ANC visits also significantly increased the number of danger signs known, except in Haiti and Malawi for clients in their first pregnancy. Other studies have also found an increase in knowledge of danger signs associated with increases in the level of client’s education and the number of ANC visits (Duysburgh et al. 2013; Kabakyenga et al. 2011; Mutiso et al. 2008; Pembe et al. 2009). Concerning training, only in Senegal did the regression results indicate that clients who saw a provider who was trained in ANC counseling had significantly more knowledge of danger signs of pregnancy complications. A study in Guatemala found that the likelihood of having heard of danger signs almost tripled after implementation of a training for clinic-based providers in prenatal counseling (Perreira et al. 2002).

One of the main limitations in examining the effect of provider counseling on the level of client knowledge of danger signs is that the client may have learned about the danger signs elsewhere rather than during the ANC visit. We attempted to control for this bias by performing a separate analysis for clients in their first pregnancy in order to capture women who might be learning about danger signs of pregnancy complications for the first time. For these clients as well as all clients, however, the regression results still showed that only when the client reported that the counseling occurred was there a significant increase in the number of danger signs known. In addition, despite the possibility that clients could have learned about danger signs elsewhere, the level of knowledge was still relatively low; as mentioned, most clients were unable to report any danger sign, or only one.

Increases both in the quantity and the quality of counseling appear required in order to increase the level of positive agreement between observation of counseling and client reports that counseling occurred. The regression analysis found that, in order to show a significant increase in knowledge of danger signs, it was important for the client to report that counseling took place. While challenges exist, providing counseling that is more client-centered and focuses on the client’s needs could improve the quality of the counseling, and as a result could increase the client’s knowledge of danger signs. Making counseling more client-centered and improving the interaction between provider and client can improve the quality of counseling (Chewning and Sleath 1996; Clift 2001; Jennings et al. 2010; Roter 2000). Health facilities in Haiti, Senegal, and Malawi should examine the content of the typical ANC consultation and work with providers to ensure that they cover the necessary services and provide essential ANC counseling, especially in danger signs of pregnancy complications.

## Data Availability

All data used in this manuscript are publically available at the DHS website: http://www.dhsprogram.com/Data/.

## Footnotes

1 The DHS Program SPA Methodology: https://dhsprogram.com/What-We-Do/Survey-Types/SPA.cfm.
